# (*E*)-Ethyl 2-anilino-5-[3-(dimethyl­amino)­acrylo­yl]-4-phenyl­thio­phene-3-carboxyl­ate

**DOI:** 10.1107/S1600536813003231

**Published:** 2013-02-06

**Authors:** Yahia Nasser Mabkhot, Assem Barakat, Fatima Alatibi, M. Iqbal Choudhary, Sammer Yousuf

**Affiliations:** a Department of Chemistry, College of Science, King Saud University, PO Box 2455, Riyadh 11451, Saudi Arabia; b Department of Chemistry, Faculty of Science, Alexandria University, PO Box 426, Ibrahimia, 21321 Alexandria, Egypt; c H.E.J. Research Institute of Chemistry, International Center for Chemical and Biological Sciences, University of Karachi, Karachi 75270, Pakistan

## Abstract

In the title compound, C_24_H_24_N_2_O_3_S, the phenyl rings form dihedral angles of 55.65 (11) and 79.60 (11)° with the plane of the thio­phene ring. The mol­ecular conformation is stabilized by an intra­molecular N—H⋯O hydrogen bond, generating an *S*(6) ring motif. In the crystal, centrosymmetrically related mol­ecules are linked into dimers by two pairs of C—H⋯O inter­actions.

## Related literature

For background to biological activity of thio­phene derivatives see: Mishra *et al.* (2011 ▶). For the synthesis of different thio­phene derivatives, see: Mabkhot *et al.* (2011 ▶); Mabkhot, Barakat & Alshahrani (2012 ▶); Mabkhot, Barakat, Al-Majid, Alamary & Al-Nahary (2012 ▶); Mabkhot, Barakat, Al-Majid & Alshahrani (2012 ▶). For related structures, see: Cao *et al.* (2003 ▶).
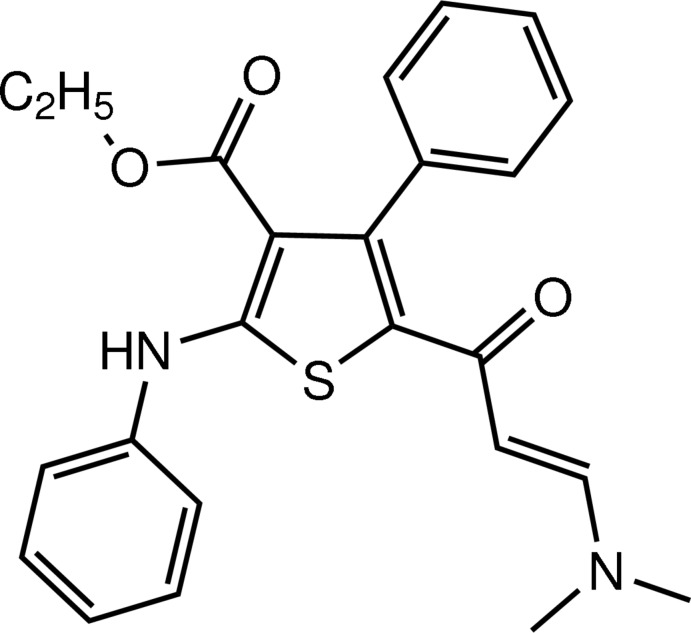


## Experimental

### 

#### Crystal data

C_24_H_24_N_2_O_3_S
*M* *_r_* = 420.51
Triclinic, 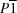

*a* = 6.5776 (9) Å
*b* = 10.7119 (14) Å
*c* = 16.516 (2) Å
α = 78.459 (3)°
β = 79.743 (3)°
γ = 80.765 (3)°
*V* = 1112.5 (3) Å^3^
*Z* = 2
Mo *K*α radiation
μ = 0.17 mm^−1^
*T* = 273 K
0.28 × 0.27 × 0.18 mm

#### Data collection

Bruker SMART APEX CCD area-detector diffractometer
Absorption correction: multi-scan (*SADABS*; Bruker, 2000 ▶) *T*_min_ = 0.953, *T*_max_ = 0.970
12618 measured reflections
4123 independent reflections
3373 reflections with *I* > 2σ(*I*)
*R*_int_ = 0.021

#### Refinement

*R*[*F* ^2^ > 2σ(*F* ^2^)] = 0.047
*wR*(*F* ^2^) = 0.142
*S* = 1.05
4123 reflections
272 parameters
H-atom parameters constrained
Δρ_max_ = 0.27 e Å^−3^
Δρ_min_ = −0.20 e Å^−3^

### 

Data collection: *SMART* (Bruker, 2000 ▶); cell refinement: *SAINT* (Bruker, 2000 ▶); data reduction: *SAINT*; program(s) used to solve structure: *SHELXS97* (Sheldrick, 2008 ▶); program(s) used to refine structure: *SHELXL97* (Sheldrick, 2008 ▶); molecular graphics: *SHELXTL* (Sheldrick, 2008 ▶); software used to prepare material for publication: *SHELXTL*, *PARST* (Nardelli, 1995 ▶) and *PLATON* (Spek, 2009 ▶).

## Supplementary Material

Crystal structure: contains datablock(s) global, I. DOI: 10.1107/S1600536813003231/rz5041sup1.cif

Structure factors: contains datablock(s) I. DOI: 10.1107/S1600536813003231/rz5041Isup2.hkl

Supplementary material file. DOI: 10.1107/S1600536813003231/rz5041Isup3.cml

Additional supplementary materials:  crystallographic information; 3D view; checkCIF report

## Figures and Tables

**Table 1:** Hydrogen-bond geometry (Å, °)

*D*—H⋯*A*	*D*—H	H⋯*A*	*D*⋯*A*	*D*—H⋯*A*
N1—H1*A*⋯O2	0.86	2.07	2.709 (3)	130
C19—H19*A*⋯O3^i^	0.93	2.42	3.294 (3)	157
C21—H21*A*⋯O3^i^	0.96	2.60	3.491 (4)	155

Symmetry code: (i) 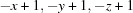.

**Fig. 1.**
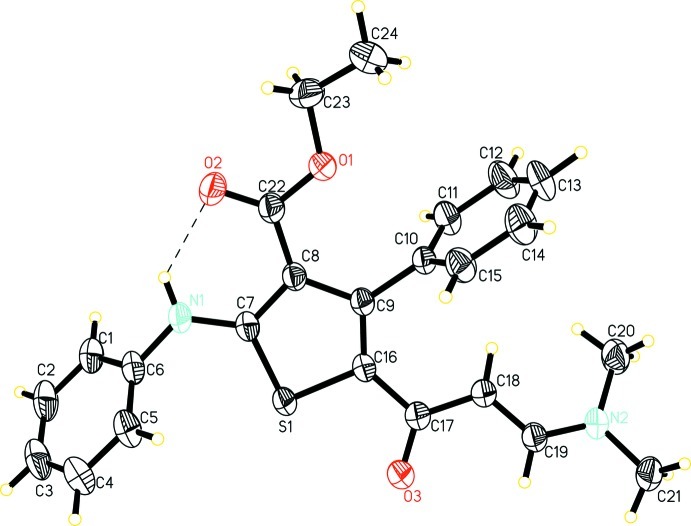
The molecular structure of title compound with displacement ellipsoids drawn at the 30% probability level. Dashed line indicates the intramolecular hydrogen bond.

**Fig. 2.**
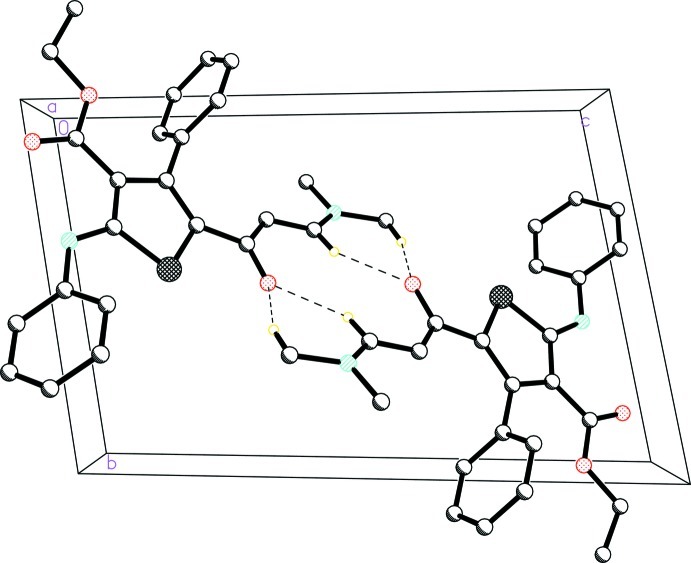
Crystal packing of the title compound viewed along the a axis. Only hydrogen atoms involved in hydrogen bonding (dashed lines) are shown.

## supplementary crystallographic information

### Comment

Thiophene moeities containing hetereocyclic compounds are well known for their wide range of biological activities such as antidiabetic, antiinflammatory, antibacterial, antidepressent and antiellergic (Mishra *et al.*, 2011). Mabkhot and co-workers have been extensively involved in the synthesis of biologically valuable thiophene derivatives (Mabkhot *et al.*, 2011; Mabkhot, Barakat & Alshahrani, 2012; Mabkhot, Barakat, Al-Majid Alamary & Al-Nahary, 2012; Mabkhot, Barakat, Al-Majid & Alshahrani, 2012). The title compound is an enaminone derivative of a substituted thiophene nucleus, and was synthesized in order to create a library for the evaluation of different biological activities.

The structure of the title compound is composed of a central thiophene unit (S1/C7–C9/C16) with an aminophenyl (N1/C1–C6), an ethyl acyl (O1–O2/C22—C24), a phenyl (C10–C15) and an enaminone (O3/N2/C17–C21) susbtituent attached to C7, C8, C9 and C16, respectively. The thiophene and phenyl rings form dihedral angles of 55.65 (11), 79.60 (11) and 24.67 (12)° between S1/C7–C9/C16 and C1–C6, S1/C7–C9/C16 and C10–C15) and C1–C6 and C10–C15, respectively. The C18–C19 (1.357 (3) Å) olefinic bond of the enaminone side chain has an *E* configuration. The shorter C—C bond length of C17–C18 (1.423 (3) Å), as compared to single bond (1.54 Å), indicates that the olefinic bond is involved in conjugation with a carbonyl functionality (C17–O3, 1.240 (2) Å). The bond lengths and angles were found to be in same range as in other related compounds (Cao *et al.*, 2003). The conformation of the molecule is stabilized by an N1—H1A···O2 intramolecular hydrogen bond to form an *S*6 graph set ring motif (Fig. 1, Table 1). In the crystal (Fig. 2), centrosymmetrically related molecules are linked *via* C19—H19A···O3 and C21—H21A···O3 intermolecular hydrogen bonds (symmetry codes as in Table 1) into dimers.

### Experimental

The title compound was synthesized by following the same procedure as described by Mabkhot *et al.*, 2011). The compound was recrystallized from a 95% ethanol solution to obtain dark yellow crystals (m. p. 440 K) found to be suitable for single-crystal X-ray data collection. All chemicals were purchased from Sigma- Aldrich.

### Refinement

H atoms on methyl, methylene, methine and nitrogen atoms were positioned geometrically with C—H = 0.93–0.97 Å, N—H = 0.86 Å, and constrained to ride on their parent atoms with *U*_iso_(H)= 1.2*U*_eq_ (CH_2_, CH, NH) or 1.5*U*_eq_(CH_3_).

### Crystal data

**Table:**

C_24_H_24_N_2_O_3_S	*Z* = 2
*M**_r_* = 420.51	*F*(000) = 444
Triclinic, *P*1	*D*_x_ = 1.255 Mg m^−^^3^
Hall symbol: -P 1	Mo *K*α radiation, λ = 0.71073 Å
*a* = 6.5776 (9) Å	Cell parameters from 4320 reflections
*b* = 10.7119 (14) Å	θ = 1.3–25.5°
*c* = 16.516 (2) Å	µ = 0.17 mm^−^^1^
α = 78.459 (3)°	*T* = 273 K
β = 79.743 (3)°	Block, yellow
γ = 80.765 (3)°	0.28 × 0.27 × 0.18 mm
*V* = 1112.5 (3) Å^3^	

### Data collection

**Table:**

Bruker SMART APEX CCD area-detector diffractometer	4123 independent reflections
Radiation source: fine-focus sealed tube	3373 reflections with *I* > 2σ(*I*)
Graphite monochromator	*R*_int_ = 0.021
ω scan	θ_max_ = 25.5°, θ_min_ = 1.3°
Absorption correction: multi-scan (*SADABS*; Bruker, 2000)	*h* = −7→7
*T*_min_ = 0.953, *T*_max_ = 0.970	*k* = −12→12
12618 measured reflections	*l* = −19→19

### Refinement

**Table:**

Refinement on *F*^2^	Secondary atom site location: difference Fourier map
Least-squares matrix: full	Hydrogen site location: inferred from neighbouring sites
*R*[*F*^2^ > 2σ(*F*^2^)] = 0.047	H-atom parameters constrained
*wR*(*F*^2^) = 0.142	*w* = 1/[σ^2^(*F*_o_^2^) + (0.0761*P*)^2^ + 0.2541*P*] where *P* = (*F*_o_^2^ + 2*F*_c_^2^)/3
*S* = 1.05	(Δ/σ)_max_ < 0.001
4123 reflections	Δρ_max_ = 0.27 e Å^−^^3^
272 parameters	Δρ_min_ = −0.20 e Å^−^^3^
0 restraints	Extinction correction: *SHELXL97* (Sheldrick, 2008), Fc^*^=kFc[1+0.001xFc^2^λ^3^/sin(2θ)]^-1/4^
Primary atom site location: structure-invariant direct methods	Extinction coefficient: 0.004 (2)

### Special details

**Table:**

Geometry. All e.s.d.'s (except the e.s.d. in the dihedral angle between two l.s. planes) are estimated using the full covariance matrix. The cell e.s.d.'s are taken into account individually in the estimation of e.s.d.'s in distances, angles and torsion angles; correlations between e.s.d.'s in cell parameters are only used when they are defined by crystal symmetry. An approximate (isotropic) treatment of cell e.s.d.'s is used for estimating e.s.d.'s involving l.s. planes.
Refinement. Refinement of *F*^2^ against ALL reflections. The weighted *R*-factor *wR* and goodness of fit *S* are based on *F*^2^, conventional *R*-factors *R* are based on *F*, with *F* set to zero for negative *F*^2^. The threshold expression of *F*^2^ > σ(*F*^2^) is used only for calculating *R*-factors(gt) *etc*. and is not relevant to the choice of reflections for refinement. *R*-factors based on *F*^2^ are statistically about twice as large as those based on *F*, and *R*- factors based on ALL data will be even larger.

### Fractional atomic coordinates and isotropic or equivalent isotropic displacement parameters (Å^2^)

**Table:**

	*x*	*y*	*z*	*U*_iso_*/*U*_eq_	
S1	0.88423 (8)	0.46635 (5)	0.24436 (3)	0.0586 (2)	
O1	0.9061 (3)	0.04813 (14)	0.16466 (10)	0.0714 (4)	
O2	1.1246 (3)	0.16911 (16)	0.07700 (11)	0.0837 (5)	
O3	0.5995 (3)	0.49861 (18)	0.38688 (12)	0.0932 (6)	
N1	1.1535 (3)	0.40198 (17)	0.11137 (11)	0.0613 (5)	
H1A	1.1971	0.3505	0.0763	0.074*	
N2	0.0937 (3)	0.31677 (18)	0.51004 (11)	0.0648 (5)	
C1	1.2694 (3)	0.5907 (2)	0.01997 (14)	0.0666 (6)	
H1B	1.2136	0.5698	−0.0228	0.080*	
C2	1.3713 (4)	0.6972 (2)	0.00528 (18)	0.0797 (7)	
H2B	1.3839	0.7481	−0.0476	0.096*	
C3	1.4536 (4)	0.7293 (2)	0.0667 (2)	0.0851 (8)	
H3A	1.5233	0.8012	0.0558	0.102*	
C4	1.4336 (4)	0.6544 (3)	0.14570 (19)	0.0851 (8)	
H4A	1.4881	0.6767	0.1883	0.102*	
C5	1.3326 (3)	0.5465 (2)	0.16131 (16)	0.0712 (6)	
H5A	1.3205	0.4955	0.2142	0.085*	
C6	1.2499 (3)	0.51465 (19)	0.09836 (14)	0.0572 (5)	
C7	0.9978 (3)	0.36762 (18)	0.17430 (12)	0.0511 (5)	
C8	0.9084 (3)	0.25406 (17)	0.18880 (11)	0.0488 (4)	
C9	0.7401 (3)	0.25055 (17)	0.25779 (11)	0.0464 (4)	
C10	0.6210 (3)	0.13903 (17)	0.28969 (11)	0.0477 (4)	
C11	0.4610 (3)	0.1211 (2)	0.25139 (14)	0.0670 (6)	
H11A	0.4247	0.1799	0.2050	0.080*	
C12	0.3543 (4)	0.0164 (3)	0.28151 (19)	0.0899 (8)	
H12A	0.2458	0.0051	0.2556	0.108*	
C13	0.4077 (5)	−0.0709 (3)	0.34945 (19)	0.0919 (9)	
H13A	0.3382	−0.1427	0.3688	0.110*	
C14	0.5630 (5)	−0.0524 (2)	0.38873 (17)	0.0837 (8)	
H14A	0.5969	−0.1105	0.4358	0.100*	
C15	0.6697 (4)	0.0519 (2)	0.35904 (14)	0.0652 (6)	
H15A	0.7758	0.0637	0.3861	0.078*	
C16	0.7077 (3)	0.35877 (18)	0.29284 (12)	0.0510 (5)	
C17	0.5610 (3)	0.4031 (2)	0.36344 (13)	0.0592 (5)	
C18	0.3834 (3)	0.33914 (19)	0.39894 (13)	0.0560 (5)	
H18A	0.3461	0.2800	0.3718	0.067*	
C19	0.2683 (3)	0.3641 (2)	0.47208 (13)	0.0578 (5)	
H19A	0.3165	0.4201	0.4984	0.069*	
C20	0.0008 (4)	0.2328 (3)	0.47236 (17)	0.0910 (9)	
H20A	0.0754	0.2277	0.4173	0.136*	
H20B	−0.1422	0.2665	0.4687	0.136*	
H20C	0.0078	0.1486	0.5061	0.136*	
C21	−0.0001 (4)	0.3365 (3)	0.59369 (16)	0.0827 (7)	
H21A	0.0754	0.3926	0.6120	0.124*	
H21B	0.0049	0.2554	0.6311	0.124*	
H21C	−0.1424	0.3745	0.5933	0.124*	
C22	0.9907 (3)	0.15623 (19)	0.13755 (13)	0.0578 (5)	
C23	0.9697 (5)	−0.0552 (2)	0.11770 (18)	0.0968 (9)	
H23A	0.9220	−0.0306	0.0637	0.116*	
H23B	1.1205	−0.0732	0.1083	0.116*	
C24	0.8828 (6)	−0.1672 (3)	0.1632 (2)	0.1179 (12)	
H24A	0.9261	−0.2363	0.1324	0.177*	
H24B	0.7335	−0.1494	0.1713	0.177*	
H24C	0.9302	−0.1910	0.2166	0.177*	

### Atomic displacement parameters (Å^2^)

**Table:**

	*U*^11^	*U*^22^	*U*^33^	*U*^12^	*U*^13^	*U*^23^
S1	0.0597 (3)	0.0517 (3)	0.0638 (3)	−0.0230 (2)	0.0070 (2)	−0.0104 (2)
O1	0.0851 (11)	0.0547 (8)	0.0723 (10)	−0.0266 (8)	0.0191 (8)	−0.0197 (7)
O2	0.0937 (12)	0.0687 (10)	0.0789 (11)	−0.0259 (9)	0.0347 (9)	−0.0200 (8)
O3	0.1033 (13)	0.0889 (12)	0.0949 (13)	−0.0538 (11)	0.0363 (10)	−0.0462 (10)
N1	0.0593 (10)	0.0572 (10)	0.0626 (10)	−0.0223 (8)	0.0135 (8)	−0.0077 (8)
N2	0.0615 (10)	0.0710 (11)	0.0586 (10)	−0.0229 (9)	0.0085 (8)	−0.0076 (9)
C1	0.0562 (12)	0.0691 (14)	0.0638 (13)	−0.0128 (10)	0.0044 (10)	0.0052 (11)
C2	0.0621 (14)	0.0681 (15)	0.0893 (18)	−0.0143 (12)	0.0089 (13)	0.0193 (13)
C3	0.0613 (14)	0.0622 (14)	0.121 (2)	−0.0241 (11)	0.0048 (15)	0.0067 (15)
C4	0.0691 (15)	0.0835 (17)	0.106 (2)	−0.0331 (13)	−0.0149 (14)	−0.0050 (15)
C5	0.0605 (13)	0.0720 (14)	0.0771 (15)	−0.0246 (11)	−0.0114 (11)	0.0097 (12)
C6	0.0418 (10)	0.0529 (11)	0.0688 (13)	−0.0110 (8)	0.0034 (9)	0.0015 (9)
C7	0.0478 (10)	0.0496 (10)	0.0522 (10)	−0.0124 (8)	−0.0020 (8)	−0.0007 (8)
C8	0.0486 (10)	0.0471 (10)	0.0484 (10)	−0.0126 (8)	−0.0014 (8)	−0.0030 (8)
C9	0.0460 (10)	0.0475 (10)	0.0444 (9)	−0.0119 (8)	−0.0062 (8)	−0.0009 (8)
C10	0.0478 (10)	0.0465 (10)	0.0466 (10)	−0.0121 (8)	−0.0008 (8)	−0.0041 (8)
C11	0.0658 (13)	0.0723 (14)	0.0640 (13)	−0.0282 (11)	−0.0150 (11)	0.0052 (11)
C12	0.0818 (17)	0.099 (2)	0.0988 (19)	−0.0515 (15)	−0.0179 (15)	−0.0043 (16)
C13	0.097 (2)	0.0732 (16)	0.103 (2)	−0.0499 (15)	0.0031 (17)	0.0069 (15)
C14	0.101 (2)	0.0623 (14)	0.0775 (16)	−0.0230 (14)	−0.0109 (15)	0.0192 (12)
C15	0.0724 (14)	0.0579 (12)	0.0632 (13)	−0.0174 (10)	−0.0158 (11)	0.0062 (10)
C16	0.0487 (10)	0.0520 (11)	0.0513 (10)	−0.0174 (8)	−0.0003 (8)	−0.0048 (8)
C17	0.0621 (12)	0.0575 (12)	0.0577 (12)	−0.0190 (10)	0.0036 (10)	−0.0117 (9)
C18	0.0569 (12)	0.0549 (11)	0.0556 (11)	−0.0150 (9)	0.0013 (9)	−0.0106 (9)
C19	0.0581 (12)	0.0532 (11)	0.0594 (12)	−0.0149 (9)	0.0022 (9)	−0.0071 (9)
C20	0.0783 (17)	0.130 (2)	0.0743 (16)	−0.0554 (17)	−0.0024 (13)	−0.0165 (16)
C21	0.0838 (17)	0.0813 (16)	0.0765 (16)	−0.0251 (13)	0.0255 (13)	−0.0201 (13)
C22	0.0592 (12)	0.0525 (11)	0.0578 (12)	−0.0138 (9)	0.0019 (10)	−0.0049 (9)
C23	0.135 (3)	0.0640 (15)	0.0864 (18)	−0.0254 (16)	0.0287 (17)	−0.0319 (13)
C24	0.144 (3)	0.0774 (19)	0.135 (3)	−0.0471 (19)	0.028 (2)	−0.0445 (19)

### Geometric parameters (Å, º)

**Table:**

S1—C7	1.716 (2)	C10—C15	1.376 (3)
S1—C16	1.7454 (18)	C10—C11	1.376 (3)
O1—C22	1.327 (2)	C11—C12	1.380 (3)
O1—C23	1.443 (3)	C11—H11A	0.9300
O2—C22	1.211 (2)	C12—C13	1.368 (4)
O3—C17	1.240 (2)	C12—H12A	0.9300
N1—C7	1.361 (2)	C13—C14	1.362 (4)
N1—C6	1.414 (3)	C13—H13A	0.9300
N1—H1A	0.8600	C14—C15	1.375 (3)
N2—C19	1.332 (3)	C14—H14A	0.9300
N2—C20	1.451 (3)	C15—H15A	0.9300
N2—C21	1.451 (3)	C16—C17	1.480 (3)
C1—C2	1.374 (3)	C17—C18	1.425 (3)
C1—C6	1.381 (3)	C18—C19	1.357 (3)
C1—H1B	0.9300	C18—H18A	0.9300
C2—C3	1.354 (4)	C19—H19A	0.9300
C2—H2B	0.9300	C20—H20A	0.9600
C3—C4	1.385 (4)	C20—H20B	0.9600
C3—H3A	0.9300	C20—H20C	0.9600
C4—C5	1.382 (3)	C21—H21A	0.9600
C4—H4A	0.9300	C21—H21B	0.9600
C5—C6	1.377 (3)	C21—H21C	0.9600
C5—H5A	0.9300	C23—C24	1.426 (4)
C7—C8	1.395 (3)	C23—H23A	0.9700
C8—C9	1.440 (2)	C23—H23B	0.9700
C8—C22	1.455 (3)	C24—H24A	0.9600
C9—C16	1.367 (3)	C24—H24B	0.9600
C9—C10	1.490 (2)	C24—H24C	0.9600
			
C7—S1—C16	91.40 (9)	C12—C13—H13A	120.1
C22—O1—C23	118.51 (17)	C13—C14—C15	120.2 (2)
C7—N1—C6	125.94 (18)	C13—C14—H14A	119.9
C7—N1—H1A	117.0	C15—C14—H14A	119.9
C6—N1—H1A	117.0	C14—C15—C10	120.6 (2)
C19—N2—C20	120.91 (19)	C14—C15—H15A	119.7
C19—N2—C21	121.6 (2)	C10—C15—H15A	119.7
C20—N2—C21	117.18 (19)	C9—C16—C17	134.44 (18)
C2—C1—C6	119.8 (2)	C9—C16—S1	112.14 (14)
C2—C1—H1B	120.1	C17—C16—S1	113.40 (14)
C6—C1—H1B	120.1	O3—C17—C18	123.6 (2)
C3—C2—C1	121.0 (2)	O3—C17—C16	116.37 (18)
C3—C2—H2B	119.5	C18—C17—C16	120.04 (18)
C1—C2—H2B	119.5	C19—C18—C17	120.38 (19)
C2—C3—C4	119.7 (2)	C19—C18—H18A	119.8
C2—C3—H3A	120.2	C17—C18—H18A	119.8
C4—C3—H3A	120.2	N2—C19—C18	126.8 (2)
C5—C4—C3	120.0 (3)	N2—C19—H19A	116.6
C5—C4—H4A	120.0	C18—C19—H19A	116.6
C3—C4—H4A	120.0	N2—C20—H20A	109.5
C6—C5—C4	119.8 (2)	N2—C20—H20B	109.5
C6—C5—H5A	120.1	H20A—C20—H20B	109.5
C4—C5—H5A	120.1	N2—C20—H20C	109.5
C5—C6—C1	119.7 (2)	H20A—C20—H20C	109.5
C5—C6—N1	121.13 (19)	H20B—C20—H20C	109.5
C1—C6—N1	119.1 (2)	N2—C21—H21A	109.5
N1—C7—C8	125.77 (18)	N2—C21—H21B	109.5
N1—C7—S1	121.90 (15)	H21A—C21—H21B	109.5
C8—C7—S1	112.31 (14)	N2—C21—H21C	109.5
C7—C8—C9	111.73 (17)	H21A—C21—H21C	109.5
C7—C8—C22	120.01 (17)	H21B—C21—H21C	109.5
C9—C8—C22	128.25 (17)	O2—C22—O1	122.38 (19)
C16—C9—C8	112.39 (16)	O2—C22—C8	124.57 (19)
C16—C9—C10	123.85 (17)	O1—C22—C8	113.04 (17)
C8—C9—C10	123.71 (16)	C24—C23—O1	109.3 (2)
C15—C10—C11	118.77 (18)	C24—C23—H23A	109.8
C15—C10—C9	119.88 (17)	O1—C23—H23A	109.8
C11—C10—C9	121.34 (17)	C24—C23—H23B	109.8
C10—C11—C12	120.3 (2)	O1—C23—H23B	109.8
C10—C11—H11A	119.8	H23A—C23—H23B	108.3
C12—C11—H11A	119.8	C23—C24—H24A	109.5
C13—C12—C11	120.2 (2)	C23—C24—H24B	109.5
C13—C12—H12A	119.9	H24A—C24—H24B	109.5
C11—C12—H12A	119.9	C23—C24—H24C	109.5
C14—C13—C12	119.8 (2)	H24A—C24—H24C	109.5
C14—C13—H13A	120.1	H24B—C24—H24C	109.5
			
C6—C1—C2—C3	0.0 (4)	C10—C11—C12—C13	−0.4 (4)
C1—C2—C3—C4	−0.6 (4)	C11—C12—C13—C14	1.8 (5)
C2—C3—C4—C5	1.0 (4)	C12—C13—C14—C15	−1.7 (5)
C3—C4—C5—C6	−0.8 (4)	C13—C14—C15—C10	0.2 (4)
C4—C5—C6—C1	0.2 (3)	C11—C10—C15—C14	1.1 (3)
C4—C5—C6—N1	177.3 (2)	C9—C10—C15—C14	−179.2 (2)
C2—C1—C6—C5	0.2 (3)	C8—C9—C16—C17	−179.4 (2)
C2—C1—C6—N1	−177.0 (2)	C10—C9—C16—C17	−1.9 (4)
C7—N1—C6—C5	52.8 (3)	C8—C9—C16—S1	−1.2 (2)
C7—N1—C6—C1	−130.1 (2)	C10—C9—C16—S1	176.24 (14)
C6—N1—C7—C8	−177.4 (2)	C7—S1—C16—C9	1.72 (16)
C6—N1—C7—S1	4.4 (3)	C7—S1—C16—C17	−179.70 (16)
C16—S1—C7—N1	176.63 (17)	C9—C16—C17—O3	168.0 (2)
C16—S1—C7—C8	−1.78 (16)	S1—C16—C17—O3	−10.2 (3)
N1—C7—C8—C9	−176.91 (18)	C9—C16—C17—C18	−13.9 (4)
S1—C7—C8—C9	1.4 (2)	S1—C16—C17—C18	167.92 (16)
N1—C7—C8—C22	4.2 (3)	O3—C17—C18—C19	−13.4 (4)
S1—C7—C8—C22	−177.48 (15)	C16—C17—C18—C19	168.64 (19)
C7—C8—C9—C16	−0.1 (2)	C20—N2—C19—C18	−2.6 (4)
C22—C8—C9—C16	178.68 (19)	C21—N2—C19—C18	171.4 (2)
C7—C8—C9—C10	−177.58 (17)	C17—C18—C19—N2	176.3 (2)
C22—C8—C9—C10	1.2 (3)	C23—O1—C22—O2	−3.0 (4)
C16—C9—C10—C15	−78.4 (3)	C23—O1—C22—C8	178.1 (2)
C8—C9—C10—C15	98.8 (2)	C7—C8—C22—O2	−6.2 (3)
C16—C9—C10—C11	101.3 (2)	C9—C8—C22—O2	175.1 (2)
C8—C9—C10—C11	−81.5 (3)	C7—C8—C22—O1	172.74 (18)
C15—C10—C11—C12	−1.0 (4)	C9—C8—C22—O1	−6.0 (3)
C9—C10—C11—C12	179.3 (2)	C22—O1—C23—C24	172.2 (3)

### Hydrogen-bond geometry (Å, º)

**Table:**

*D*—H···*A*	*D*—H	H···*A*	*D*···*A*	*D*—H···*A*
N1—H1*A*···O2	0.86	2.07	2.709 (3)	130
C19—H19*A*···O3^i^	0.93	2.42	3.294 (3)	157
C21—H21*A*···O3^i^	0.96	2.60	3.491 (4)	155

Symmetry code: (i) −*x*+1, −*y*+1, −*z*+1.
